# The Effects of Fish Oil on Cardiovascular Diseases: Systematical Evaluation and Recent Advance

**DOI:** 10.3389/fcvm.2021.802306

**Published:** 2022-01-05

**Authors:** Jia Liao, Qingsong Xiong, Yuehui Yin, Zhiyu Ling, Shaojie Chen

**Affiliations:** ^1^Department of Cardiology, The Second Affiliated Hospital of Chongqing Medical University (CQMU), Chongqing, China; ^2^Cardioangiologisches Centrum Bethanien (CCB)/Kardiologie, Medizinische Klinik III, Agaplesion Markus Krankenhaus, Akademisches Lehrkrankenhaus der Goethe-Universität Frankfurt am Main, Frankfurt am Main, Germany

**Keywords:** fish oil, ω-3 PUFAs, cardiovascular disease, hypertension, coronary heart disease, atrial fibrillation, heart failure, arrhythmia

## Abstract

Fish oil is rich in unsaturated fatty acids, i.e., eicosapentaenoic acid (EPA) and docosahexaenoic acid (DHA), both of which are widely distributed in the body such as heart and brain. *In vivo* and *in vitro* experiments showed that unsaturated fatty acids may have effects of anti-inflammation, anti-oxidation, protecting vascular endothelial cells, thrombosis inhibition, modifying autonomic nerve function, improving left ventricular remodeling, and regulating blood lipid. Given the relevance to public health, there has been increasing interest in the research of potential cardioprotective effects of fish oil. Accumulated evidence showed that fish oil supplementation may reduce the risk of cardiovascular events, and, in specific, it may have potential benefits in improving the prognosis of patients with hypertension, coronary heart disease, cardiac arrhythmias, or heart failure; however, some studies yielded inconsistent results. In this article, we performed an updated systematical review in order to provide a contemporary understanding with regard to the effects of fish oil on cardiovascular diseases.

## Introduction

Fish oil is rich in long-chain omega-3 polyunsaturated fatty acids (ω-3 PUFAs), which mainly consist of eicosapentaenoic acid (EPA) and docosahexaenoic acid (DHA). The earliest publications of PUFAs were in the late 1970s when researchers attributed the low incidence of coronary heart disease (CHD) in Denmark to their traditional marine diet (mammals and fish) (1, 2). The first study was conducted in Greenland Inuit, in which the investigators identified the association of higher EPA and DHA intake and lower mortality from myocardial infarction (MI) and ischemic heart disease (3). Later, there has been an increasing number of studies showing the correlation between the intake of PUFAs and reduced risk of cardiovascular diseases (CVDs), i.e., CHD, MI, hypertension, arrhythmias, and heart failure (HF). These promising results have attracted extensive research to investigate the potential biological mechanisms underlying the cardiovascular protective effect of ω-3 PUFAs. *In vivo* and *in vitro* studies have demonstrated that ω-3 PUFAs may have multiple physiological functions such as anti-inflammation, anti-oxidation, protecting vascular endothelial cells, thrombosis inhibition, modifying autonomic nerve function, improving left ventricular remodeling, and regulating blood lipid. In this article, we performed an updated systematical review in order to give a contemporary understanding with regard to the effects of fish oil on CVDs.

## Source and Metabolism of ω-3 PUFAs

Fatty acids can be divided into long chain fatty acids (containing 14–24 carbon), medium chain fatty acids (8–12 carbon), and short chain fatty acids (<6 carbon). According to the degree of saturation, they can be divided into saturated fatty acids (no unsaturated double bonds in the carbon chain) and unsaturated fatty acids (containing one or more unsaturated double bonds). Unsaturated fatty acids can be divided into ω-3 series, ω-6 series, and ω-9 series according to the position of their double bonds. α-linolenic acid (ALA; 18:3n-3) and linoleic acid (LA; 18:2n-6) are ω-3 and ω-6 PUFAs' short chain acid, respectively. With a series of desaturase and carbon chain extension enzyme reactions, ALA is mainly converted into EPA (20:5n-3) and DHA (22:6n-3); LA is mainly converted to arachidonic acid (AA; C20:4n-6). In recent decades, due to the increased intake of LA, ω-6 PUFAs can meet the needs of the body. While because LA competitively inhibits the conversion of ALA to EPA and DHA, and ALA itself is rarely consumed, the transformation of ω-3 PUFAs in the body is still seriously insufficient which cannot meet the basic needs (4). Dietary ω-3 PUFAs can be obtained from animal or plant sources. Both EPA and DHA are mostly found in sea fishes, while ALA is commonly contained in Perilla oil and linseed oil. The proportion of ALA converted to EPA and DHA is usually about 5% and <1%, respectively (5).

**Figure 1:**
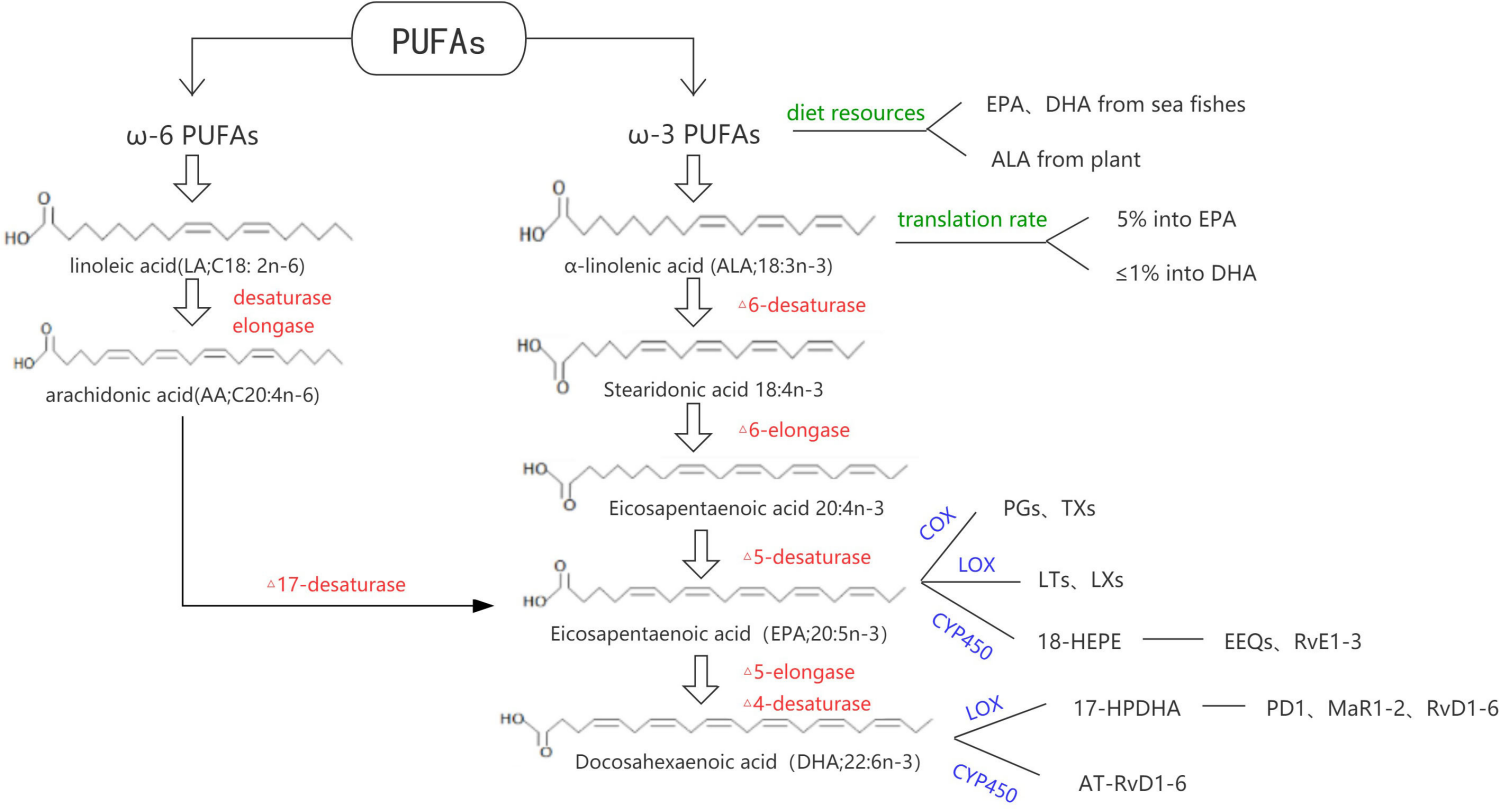
Metabolic pathway of PUFAs.

In the metabolic process of ω-3 PUFAs (Figure 1), three major oxidation enzyme systems play an important role: Cyclooxygenase (COX) and subsequent synthases, Lipoxygenase (LOX), and Cytochrome P450 (CYP450). Classic oxylipins from ω-3 PUFAs include 3-series prostaglandins (PGs), thromboxanes (TXs), 5-series leukotrienes (LTs), 5-series lipoxins (LXs), epoxyeicosatetraenoic acids (EEQs), and epoxydocosapentaenoic acids (EDPs). Some oxylipins have a similar structure and usually participate in the resolution of inflammation, which designated specialized pro-resolving mediators (SPMs) including resolvins, protectins, and maresins. DHA can be converted to 17-peroxide docoapentaenoic acid (17-HPDHA) under the action of the corresponding LOX, and then further metabolized into protectin D1 (PD1), D-series resolvins (RvD1-D6), and maresins (MaR1-MaR2). In another way, DHA can be converted to aspirin-triggered resolving D (AT-RvD1-6). On the other hand, EPA can be converted to 18-hydroxyl eicosapentaenoic acid (18-HEPE) by acetylation of the cycooxidase-2 or CYP450, and then metabolized into the E-series resolvins (RvE1-3) (5–7).

## Potential Molecular Mechanisms for Cardiovascular Protective Effect (Evidence from Basic Researches)

### Anti-inflammation

**Figure 2:**
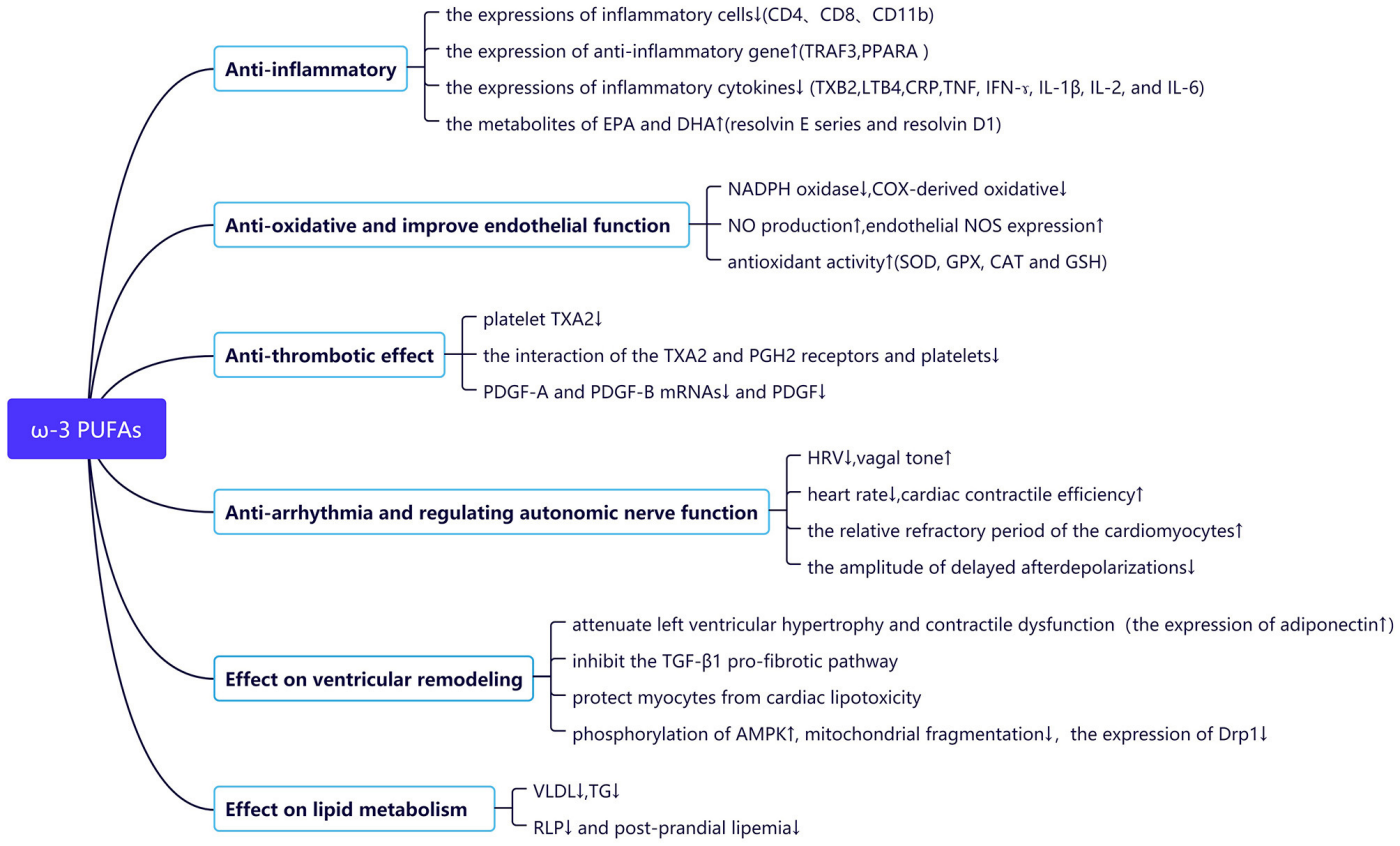
Potential molecular mechanisms for cardiovascular protective effects of the ω-3 PUFAs.

Studies in healthy subjects and cardiovascular high-risk patients suggested that a supplement of ω-3 PUFAs may be an effective treatment to reduce inflammation (8–10) (Figure 2). The resolvin E series is synthesized by EPA, which can effectively reduce the tracking of leukocytes to inflammatory sites, promote the clearance of inflammatory cells, and inhibit the production of cytokines (11). Resolvin D1 synthesized by DHA may induce the transformation of anti-inflammatory M2 macrophages, which reduced the pro-fibrotic genes and decreased collagen deposition, thereby reducing post-MI fibrosis and, thus, stabilizing the extracellular matrix (12). In another way, studies showed that ω-3 PUFAs may suppress the expressions of inflammatory cells (CD4, CD8, and CD11b) (13) and inflammatory cytokines (C-reactive protein (CRP), tumor necrosis factor (TNF), interferon (IFN-γ), interleukin (IL-1β, IL-2, and IL-6) (10, 13), and enhance the expression of anti-inflammation gene expression [peroxisome proliferator-activated receptor alpha (PPARA) and TNF receptor associated factor 3 (TRAF3)] (8). A meta-analysis demonstrated the beneficial effects of ω-3 PUFAs on reducing the concentration of TXB2 in blood and LTB4 in neutrophils (14).

### Anti-oxidation and Improved Endothelial Function

Animal and clinical studies have shown that ω-3 PUFAs may have an anti-oxidative effect and may improve endothelial function in different ways (Figure 2). Intake of EPA: DHA 6:1 prevented Ang II-induced hypertension and endothelial dysfunction by improving both the nitric oxide (NO) and endothelium-dependent hyperpolarization (EDH) mediated relaxations, preventing NADPH oxidase and COX-derived oxidative stress (15). In Apo E knockout mice, the fish oil-rich diet increased NO production and endothelial NO synthase (NOS) expression and lowered the expression of p22 phlox and O2-generation (16). Moreover, ω-3 PUFAs may increase antioxidant activity, such as superoxide dismutase (SOD), guaiacol peroxidase (GPX), catalase (CAT), and glutathione (GSH), enhancing the resistance to free radical attack and reducing lipid peroxidation and oxidative stress (17–19). Diet with a 1:1 ratio of EPA/DHA improved the oxidative stress parameters (SOD and GPX in erythrocytes) and plasma antioxidant capacity (19).

### Anti-thrombotic Effect

EPA and DHA may prevent platelet-involved thrombosis formation (Figure 2). Thromboxane is considered to be an important vector in the process of platelet aggregation (20). Previous studies showed that EPA and DHA may inhibit the interaction of the TXA2 and PGH2 receptors of platelets, and suppress the synthesis of platelet TXA2 (21). Dietary ω-3 PUFAs have been shown to downregulate platelet-derived growth factor (PDGF)-A and B-chain mRNAs, leading to reduced levels of PDGF (22).

### Anti-arrhythmia and Regulating Autonomic Nerve Function

Experimental studies have shown that fish oil may exert its antiarrhythmic effect by the direct influence of a cardiac electrophysiological character or indirect regulation of autonomic nerve function (23–25) (Figure 2). ω-3 PUFAs may inhibit sarcolemmal ion channels, stabilize electrical activity, and prolong the relative refractory period of the cardiomyocytes (23). Moreover, EPA may dose dependently reduce pulmonary vein spontaneous activity and the amplitude of delayed afterdepolarizations in rabbit tissue (25). On the other hand, heart rate variability (HRV) is a surrogate index of autonomic nerve function. Low HRV may reflect a decrease in vagal activity or responsiveness of the heart and can be a predictor of sudden cardiac death in patients with ischemic heart disease (26). A meta-analysis of 15 studies showed that short-term fish-oil supplementation may favorably influence the frequency domain of HRV, as a possible consequence of enhanced vagal tone (24). Other meta-analyses demonstrated that ω-3 PUFAs supplementation may reduce heart rate primarily by DHA rather than by EPA (27, 28). An animal study showed that supplementing diets with high-dose fish oil may enhance cardiac contractile efficiency and improve cardiac function (29).

### Effect on Ventricular Remodeling

Cardiac ventricular dysfunction with subsequent HF is often secondary to ventricular interstitial fibrosis and remodeling and constitutes the final common pathway in a spectrum of cardiac disorders. Recent studies have shown that fish oil may improve ventricular remodeling (Figure 2). Dietary supplementation with ω-3 PUFAs may attenuate pressure-overload-induced left ventricular hypertrophy and contractile dysfunction *via* upregulation of adiponectin expression in adipose tissue and plasma (30, 31). In a rodent pressure-overloaded HF model, EPA inhibited the transforming growth factor-β1 (TGF-β1) pro-fibrotic pathway *via* free fatty acid receptor 4(FFR4), affected cardiac fibroblasts, and suppressed cardiac fibrosis without the requirement of EPA localization into the cellular membrane (32). Cardiac lipotoxicity can worsen cardiac function through excess serum free fatty acids and cause HF by chronic stimulation of adrenergic activity (33). Emerging evidence suggested that EPA may activate phosphorylation of AMP-activated protein kinase (AMPK), suppress mitochondrial fragmentation, and reduce the expression of the dynamin-related protein-1(Drp1), protecting myocytes from lipotoxicity (34).

### Effect on Lipid Metabolism

Lipid metabolism disorder as an important part of atherosclerosis progression may also be regulated by fish oil (Figure 2). ω-3 PUFAs may decrease the activity of sterol receptor element-binding protein-1c, which is the key factor in controlling lipogenesis, resulting in the reduction of very low-density lipoprotein (VLDL) and triglyceride (TG) (35, 36). Furthermore, fish oil has been shown to reduce the remnant lipoproteins (RLP) and post-prandial lipemia after fatty meals in patients with hyperlipidemic (37). It is shown that RLP can upregulate Rho-kinase in coronary vascular smooth muscle cells (VSMCs) and markedly enhance coronary vasospastic activity in patients with sudden cardiac death (38), and post-prandial increase in RLP concentrations is an independent risk factor for restenosis after successful percutaneous coronary intervention (PCI) (39).

## ω-3 PUFAs and Cardiovascular Disorders

### Fish Oil and Hypertension

#### Hypertension With Obesity, Diabetes, or Dyslipidemia (Secondary Prevention)

*In vitro* and *in vivo* studies showed that ω-3 PUFAs can protect vascular cells (endothelial cells and VSMCs) and attenuate the proinflammatory reactions in hypertension (9). In the past decades, ω-3 PUFAs have been studied in different patients with hypertension such as patients with obesity, diabetes, hyperlipidemia, pregnancy, or hypertensive children. Among overweight school children with metabolic syndrome, after receiving daily supplementation with 2.4 g of ω-3 PUFAs for 1 month, both systolic blood pressure (SBP) and diastolic blood pressure (DBP) levels were significantly decreased (40). In patients with hypertensive diabetes, previous studies showed that neither EPA nor DHA had significant effects on reduction of BP, glycated hemoglobin, fasting insulin, or C-peptide, but may tend to increase fasting glucose (41, 42). In hypertensive patients with hypertriglyceridemia, supplementation of ω-3 PUFAs formulation (1,000 mg/day, including EPA 312 mg and DHA 202 mg) significantly reduce DBP (from 81.6 ± 5.3 mmHg to 79.3 ± 5.2 mmHg, *P* < 0.05) and alleviate hypertension-related symptoms after 12 weeks (43). It has also shown that EPA may reduce cardiac afterload by decreasing vascular reflected waves and central SBP (44).

Large-sized meta-analyses have been performed based on previous clinical studies. Miller (45) conducted a meta-analysis of 70 RCTs, which stated compared with placebo, EPA+DHA reduced SBP by −1.52 mmHg (95% CI = −2.25 to −0.79) and DBP by −0.99 mmHg (95% CI = −1.54 to −0.44). The strongest effect was observed among patients with untreated hypertension (SBP = −4.51 mmHg, 95% CI = −6.12 to −2.83; DBP = −3.05 mm Hg, 95% CI = −4.35 to −1.74). AbuMweis (46) analyzed 50 clinical trials, which showed that ω-3 PUFAs supplements led to significant reductions of SBP/DBP by 2.195/1.08 mmHg, respectively, in patients with hypertension.

#### Normotensive Population (Primary Prevention)

Following initial observations, many studies have also examined the possible effect of ω-3 PUFAs as primary prevention of hypertension in normotensive subjects. INTERMAP (47) was an international cross-sectional epidemiologic study including 2,036 healthy adults from 17 population-based samples in China, Japan, and the United Kingdom, and the study reported a reduction of −1.01 mmHg SBP and −0.98 mmHg DBP with two standard deviation (SDs) higher dietary ω-3 PUFAs intake. In another study, which excluded individuals with CVDs, diabetes, or a body mass index (BMI) ≥35 kg/m^2^, as compared with individuals in the lowest ω-3 index quartile, individuals in the highest ω-3 index quartile had a significant reduction of SBP/DBP by 4/2 mmHg, respectively (*P* < 0.01) as assessed by 24 h BP monitoring (48). Prospective cohort studies have also examined the possible influence of the dietary content with ω-3 PUFAs on the development of hypertension in normotensive subjects. Danaei et al. conducted a meta-analysis, which included more than 56,000 participants. During 3–20 years follow-up, normotensive subjects with the highest dietary consumption of ω-3 PUFAs had a 27% lower risk of developing hypertension than those with the lowest intake of ω-3 PUFAs (49). Considering the high prevalence of hypertension in the general population, diets rich in ω-3 PUFAs may be a strategy for primary prevention of hypertension (50).

#### Childhood Hypertension (CH) and Pregnancy Induced-Hypertension

Impaired fetal growth is an independent cardiovascular risk factor and is associated with arterial wall thickness in children, and ω-3 PUFAs supplementation has been shown to prevent arterial wall remodeling (51, 52). Recent data analysis of 354 participants with reduced birth weight aged 8–15 years showed that, as compared with children with the lowest tertile of intake, those who had the highest tertile of dietary EPA and DHA intake had significantly lower SBP (−4.9 mmHg, 95% CI: −9.7 to −0.1) and pulse pressure (−7.7 mmHg, 95% CI: −15.0 to −0.4) (53). A cross-sectional study found that the childhood prehypertension and hypertension prevalence rates were 7.8 and 9.15%, and a regular seafood-rich dietary pattern with high ω-3 PUFAs contents was significantly associated with a reduction in BP in hypertensive children and a decrease in the risk of CH in normotensive children (54). A recent study indicated that maternal DHA intake with 600 mg during pregnancy appeared to mitigate the association between childhood overweight condition/obesity and BP (SBP:104.28 vs. 100.34 mmHg; DBP:64.7 vs. 59.76 mmHg) (55).

Many studies have shown that ω-3 PUFAs supplements for pregnant women may provide protective effects on both mother and fetus. The first study on this topic included over 5,000 women, the experimental group received ω-3 PUFAs supplements for about 20 weeks; as compared with the control group, the risk of preterm birth and preeclampsia was decreased by 20.4 and 31.5%, respectively, among participants in experimental group (56). Further research indicated that a 1% enhancement in ω-3 PUFAs levels at mid-gestation in Asian pregnant women was associated with about 24% lower risk of pregnancy-induced hypertension (57). In a recent meta-analysis of 14 studies, ω-3 PUFAs supplementation starting at week 12–33 with a mean dose of 0.2–4 g/d had a protective effect on the risk of preeclampsia [Risk Ratio (RR): 0.82; 95% CI = 0.70-0.97] (58).

**Figure 3:**
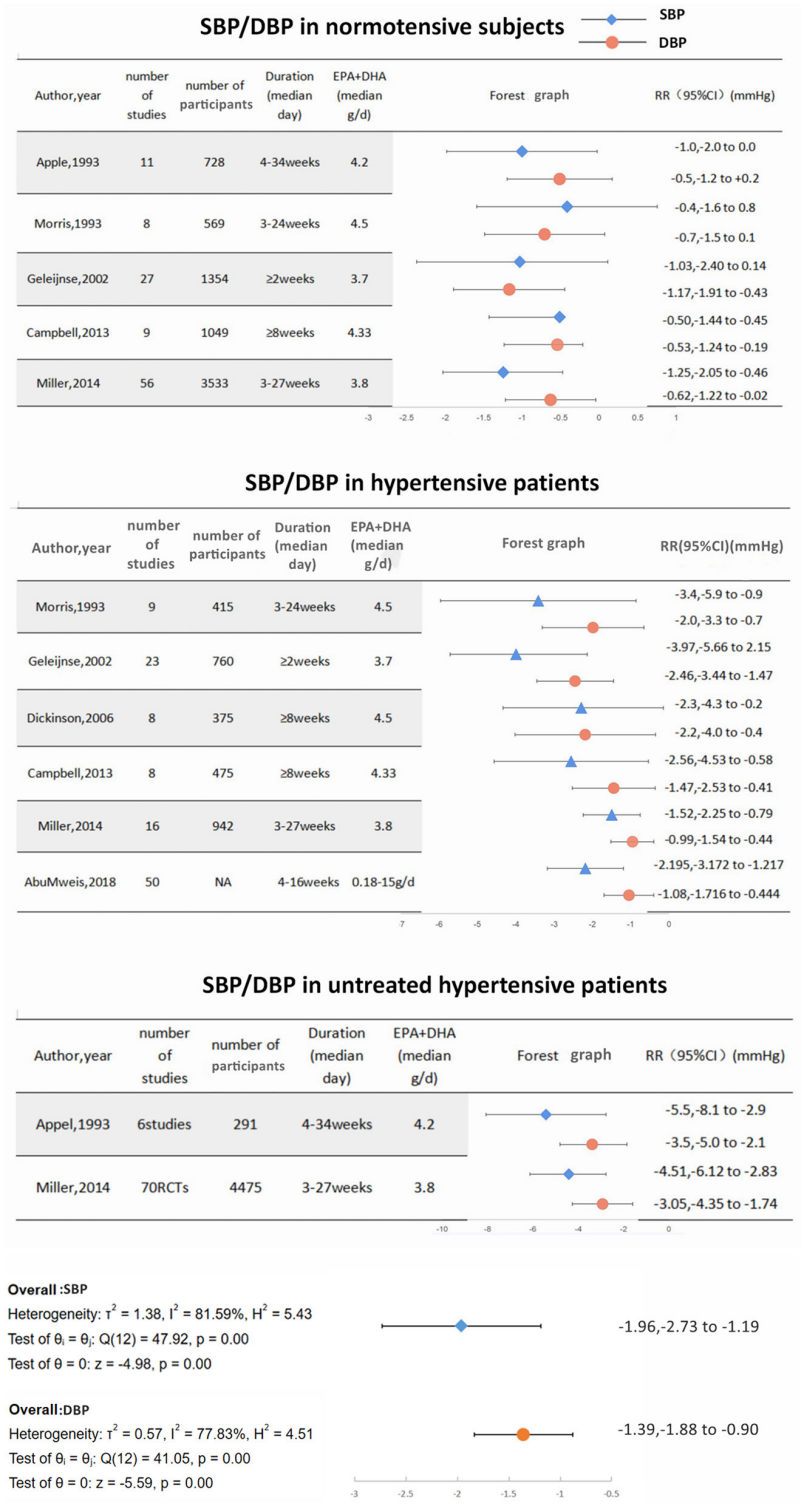
The effect of ω-3 PUFAs consumption on blood pressure in normotensive subjects/ patients with hypertension/ patients with untreated hypertension.

In summary, current evidence shows that consumption of ω-3 PUFAs is associated with a reduction in BP in different hypertensive populations and may lower the risk of developing hypertension in normotensive subjects. However, the BP lowering effect seems to be mild (SBP/DBP decreased by 3.97–1.52 mmHg/2.46–0.99 mmHg in patients with hypertension, SBP/DBP decreased by 5.5–4.51 mmHg/3.5–3.05 mmHg in patients with untreated hypertension, SBP/DBP decreased by 1.25–0.4 mmHg/1.17–0.5 mmHg in normotensive subjects) (Figure 3). The extension of the BP lowering effect appears to be dependent on the baseline BP. Moreover, no dose-dependent relationship was observed in those studies. Higher baseline BP or lower baseline ω-3 PUFAs level may predict a more significant reduction in BP after taking ω-3 PUFAs supplement. As for CH and pregnancy induced-hypertension, small studies and meta-analyses have suggested that fish oil may have a protective antihypertensive effect.

### Fish Oil and CHD

#### Primary Prevention of CHD

In patients with CHD, primary prevention is mainly to control the risk factors of CHD and guide the patients to establish a healthy lifestyle. Low-dose ω-3 PUFAs (≤1 g per day) was the initial dose used in many randomized controlled trials (RCTs). The Risk and Prevention study investigated the efficacy of ω-3 PUFAs in 12, 531 patients with multiple cardiovascular risk factors or atherosclerotic vascular disease but not MI. The experimental group received one capsule daily containing 1g ω-3 PUFAs (ratio of EPA: DHA from 0.9:1 to 1.5:1), as compared with control group receiving olive oil, there was no difference in the primary endpoint of fatal or non-fatal coronary events and death from coronary cause during 5 years follow up (59). But the results of this study may not be generalizable due to the Mediterranean dietary habits in the Italian population (60). The ORIGIN and the ASCEND trial focused on the effects of ω-3 PUFAs supplementation on cardiovascular events in patients with prediabetes and diabetes but without evidence of atherosclerotic CVD. Both studies assigned more than 10,000 patients and treated with 1 g/d for more than 5 years following up, and both studies failed to decrease the death from cardiovascular causes (RR = 0.98; 95% CI = 0.87–1.10) or the risk of serious vascular event or revascularization (RR:1.00; 95% CI = 0.91–1.09), respectively (61, 62). After that, the VITAL trial administering vitamin D3 (2,000 IU/d) and ω-3 PUFAs (1 g/d) assessed the effect of ω-3 PUFAs as primary prevention for CHD and cancer; the study found no significant association between daily consumption of (ω-3 PUFAs + vitamin D3) and major composite cardiovascular events or death from cardiovascular causes, however, subjects in (ω-3 PUFAs + vitamin D3) group had a significantly lower incidence of total MI (RR: 0.72; 95% CI: 0.59–0.9), CHD (RR: 0.83; 0.71–0.97), PCI (RR: 0.78; 95% CI: 0.63–0.95), and death from MI (RR: 0.5; 95% CI = 0.26–0.97) during 5.3 years follow-up (63).

Based on these neutral results above, it is argued that the inadequate daily dose of ω-3 PUFA (<1,000 mg) may be insufficient to take effect (64). Then the high-dose ω-3 PUFAs (>1 g per day) became the subject of fish oil research. JELIS was the first trial to investigate 1.8 g/d of purified EPA plus statin therapy compared with statin therapy alone in 18,645 patients with total cholesterol of 6.5 mmol/L or greater. Over 4.6 years, EPA treatment reduced major coronary events [including sudden cardiac death, fatal and non-fatal MI, unstable angina, and coronary artery bypass graft (CABG) or percutaneous transluminal coronary angioplasty (PTCA)] by 18% in patients with no history of coronary artery disease (65). Although nearly all patients were on a statin, only low-intensity therapy (60% were on pravastatin 10 mg daily and 36% were on simvastatin 5 mg daily) was used in accordance with Japanese practice guidelines (65). Thereafter, the REDUCE-IT (66) and STRENGTH study (67) raised the dosage to 4 g daily in patients with high cardiovascular risk, hypertriglyceridemia, and low levels of high-density lipoprotein cholesterol (HDL-C); however, the results showed the opposite outcome. In the REDUCE-IT trial, the supplement of icosapent ethyl at 2 g twice daily reduced the primary composite end point of cardiovascular death, non-fatal MI, non-fatal stroke, coronary revascularization, and unstable angina in the primary-prevention cohort (RR 0.88; 95% CI = 0.7–1.1) over 4.9 years (66). The STRENGTH study administered a combination of DHA and EPA carboxylic and the study was prematurely discontinued after a median follow-up of 3.5 years due to non-significance. None of the components of the primary outcome, or all-cause death, were significantly reduced by ω-3 PUFAs (67).

A meta-analysis of 19 cohort studies including 45,637 individuals was conducted to examine the relationship between EPA or DHA concentrations (such as plasma, serum, red blood cells, or adipose tissue) in healthy adults at the start of the study and the risk of developing CHD (68). In continuous (per 1-SD increase) multivariable-adjusted analyses, the DHA was associated with a lower risk of fatal CHD (RR: 0.90, 95% CI = 0.84–0.96). But the total CHD was not decreased by EPA (RR: 0.94; 95% CI = 0.87–1.02) and DHA (RR: 0.95; 95% CI = 0.91–1.00). More recently, a large prospective cohort study consisting of 4,27,678 health individuals who had no CVD at baseline was conducted; 31.2% of participants had habitual use of fish oil supplements, leading to significantly reduced risk of all-cause mortality (HR: 0.87; 95% CI = 0.83–0.90), CVD mortality (HR: 0.84; 95% CI = 0.78–0.91), MI incidence (HR: 0.92; 95% CI = 0.88–0.96), and stroke (HR: 0.90; 95% CI = 0.84–0.97) during long-term follow-up (at least 8–12 years) (69).

**Figure 4:**
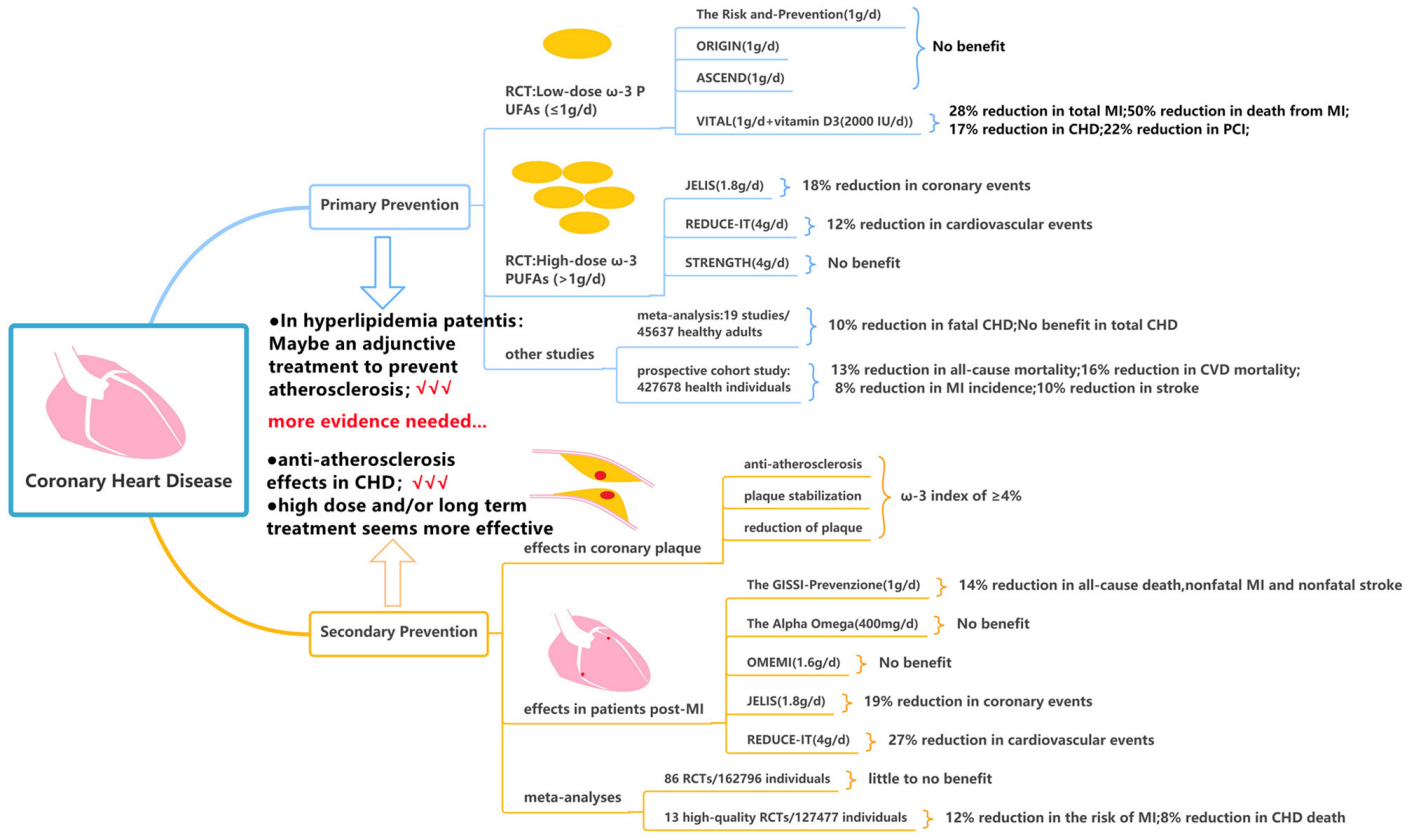
Fish oil and the primary/secondary prevention of coronary heart disease.

Based on the current evidence, the majority of published RCTs have not shown a certain benefit with ω-3 PUFAs as primary prevention of CHD in patients with cardiovascular risk factors such as age ≥65 years, male sex, hypertension, hypercholesterolemia, diabetes, current smoker, obesity, or a family history of premature CVD. But in patients with elevated triglyceride and cholesterol levels, purified EPA may serve as an adjunctive treatment in the context of statin therapy and may have a synergistic effect to lower the triglyceride and cholesterol levels and possibly further prevent atherosclerosis (Figure 4).

#### Secondary Prevention of CHD

The goal of secondary prevention is to allow an individual to correct maladjustment, restore stability, and reduce mortality and disability rates by improving and alleviating the symptom. Alfaddagh et al. found that 1.86 g of EPA and 1.5 g of DHA daily provided additional benefit to statins in preventing progression of fibrous coronary plaque in subjects adherent to therapy with well-controlled low-density-lipoprotein cholesterol (LDL-C) levels (70). The ω-3 index was calculated as the percentage of EPA and DHA of total fatty acid level. In non-diabetic subjects with an average LDL-C <80 mg/dl, an ω-3 index of ≥4% prevented progression of coronary plaques, whereas patients with an ω-3 index <3.43% were at risk of developing coronary plaques despite receiving statins (71). In patients with CHD undergoing PCI, EPA treatment may stabilize plaque assessed by optical frequency domain imaging (72) and may reduce coronary plaque volume analyzed by integrated backscatter intravascular ultrasound (73). The EVAPORATE (74) trial evaluated the effect of icosapent ethyl 2 g two times daily on low-attenuation plaque (LAP) volume *via* coronary CT angiography in 80 patients with CHD and an elevated TG level. During 18 months, ω-3 PUFAs may slow down the progression of total non-calcified plaque (sum of LAP, fibrofatty, and fibrous plaque) (35 vs. 43%, *P* = 0.01), total plaque (non-calcified + calcified plaque) (15 vs. 26%, *P* = 0.0004), fibrous plaque (17 vs. 40%, *P* = 0.011), and calcified plaque (−1 vs. 9%, *P* = 0.001). Those aforementioned studies indicated the anti-atherosclerosis effects of ω-3 PUFAs and provided important mechanistic data with regards to reducing CV events in patients with known CHD.

The GISSI-Prevenzione trial, as a large-scale RCT of ω-3 PUFAs, was conducted in 11,324 patients in Italy with recent MI (75). Supplementation with ω-3 PUFAs 1 g daily reduced the primary composite end point of all-cause death, non-fatal MI, and non-fatal stroke (RR 0.86, 95% CI = 0.74–0.99) over 3.5 years (75, 76). One limitation of the GISSI-Prevenzione trial was that the ω-3 PUFAs were administered prior to the standard use of statin therapy post-MI; consequently, only 5% of patients were on cholesterol-lowering drugs at baseline, and the number was only increased to 46% throughout the follow-up period (77). This may explain why the positive results of the GISSI-Prevenzione trial have not been replicated in recent clinical trials. The Alpha Omega trial assigned 4,837 patients with MI with a daily intake of 400 mg of ω-3 PUFAs for 40 months and it showed that low dose of ω-3 PUFAs did not significantly reduce the rates of cardiovascular end point among patients who had MI and received state-of-the-art antihypertensive, anti-thrombotic, and lipid-lowering therapy (78). Further, the OMEMI trial studied elderly patients who have survived after AMI and were treated with contemporary post-MI therapies (including dual-antiplatelet therapy and high-intensity statin) and adding on the higher dose of 1.8 g ω-3 PUFAs (930 mg EPA and 660 mg DHA) (79). During 2 years follow-up, ω-3 PUFAs did not reduce the primary composite outcome of all-cause death, non-fatal MI, stroke, unscheduled revascularization, and HF hospitalization compared with placebo (RR 1.07; 95% CI = 0.82–1.40). On the contrary, significant results were obtained in some other RCTs such as the JELIS trial and REDUCE-IT trial. The REDUCE-IT trial involving patients with established CVD or with diabetes and other risk factors (70.7% for secondary prevention of cardiovascular events) showed that the supplement of icosapent ethyl 4 g/d reduced the primary composite end point of cardiovascular death, non-fatal MI, non-fatal stroke, coronary revascularization, and unstable angina (RR 0.73; 95% CI = 0.65–0.81) (66). The JELIS trial showed that in patients with a history of CHD, who received EPA treatment, major coronary events were reduced by 19% (65).

Several meta-analyses have assessed the effect of ω-3 PUFAs on cardiovascular outcomes. Abdelhamid et al. conducted a meta-analysis of 86 RCTs (including the STRENGTH trial) evaluating ω-3 PUFAs for primary and secondary prevention of CVD with a dosage of 0.5–5 g/d. The data showed that increasing ω-3 PUFAs may reduce CHD mortality and CHD events during 12–88 months based on low-quality evidence and may have little to no effect on all-cause mortality, cardiovascular mortality, and cardiovascular events based on moderate to high-quality evidence (80). Hu et al. analyzed data from 13 high-quality RCTs, including GISSI-Prevenzione, JELIS, GISSI-HF, Alpha Omega, Omega, ORIGIN, VITAL, ASCEND, and REDUCE-IT trials, but without STRENGTH trial, and the results showed that ω-3 PUFAs supplementation lowered the risk of MI (RR 0.88, 95% CI = 0.83–0.94, *I*^2^ = 51%), CHD death (RR 0.92, 95% CI = 0.86–0.98, *I*^2^ = 21%), total CHD (RR: 0.95, 95% CI = 0.91–0.99, *I*^2^ = 35%), and CVD death (RR 0.92, 95% CI = 0.88–0.97, *I*^2^ = 6%) (81). These favorable results remained significant even after exclusion of the REDUCE-IT trial, and the risk reduction appeared to be linearly related to ω-3 PUFAs dose (81).

Based on the current evidence, fish oil seems to have effects of anti-atherosclerosis, plaque stabilization, and reduction of plaque. As secondary prevention of CHD, fish oil may be associated with improved clinical outcomes among patients with suboptimal medical treatment; however, the additional role of fish oil among patients already receiving optimal treatment remains to be determined. It seems that the protective effect of fish oil as secondary prevention of CHD appears to be dose and temporal dependent, e.g., significantly favorable results tended to be obtained from studies using high dose and/or long-term treatment, however, the optimal dose and the treatment duration remains to be defined (Figure 4).

#### Interpretation of the Controversial Results in Recent RCTs

The controversial results in recent RCTs about fish oil may stem from differences in the formulation of ω-3 PUFAs and dose of EPA and DHA, the different baseline levels of EPA and DHA, and different placebo groups (82).

The STRENGTH trial used the carboxylic acid compound as free fatty acid with enhanced oral bioavailability (67). The rapid interaction of the free fatty acid formulation with the intestinal mucosa may cause gastrointestinal adverse effects as reported in the treatment arm relative to placebo (24.7 vs. 14.7%). On the other hand, the REDUCE-IT trial (66) used the ethyl ester formulation with a slower and more controlled release of fatty acids.

Compared with EPA, DHA has an additional double bond and two more carbons that may be linked to greater susceptibility toward isomerization and reduced antioxidant activity (83). As it is known, positive outcomes were observed in the JELIS (65), REDUCE-IT (66), and EVAPORATE (74) trials in which all used EPA alone, whereas negative outcomes were observed in the STRENGTH (67), OMEMI (79), VITAL (63), and ASCEND (62) trials in which all used a mixture of EPA and DHA.

It appears that there is a relationship between the increased levels of EPA and the prognosis of CVs. In the STRENGTH trial (67), ω-3 PUFAs treatment substantially raised both plasma and red blood cell membrane concentrations of EPA (by 269 and 299%, respectively) and DHA (by 40 and 24%, respectively). On contrary, in the REDUCE-IT trial (66), the serum level of EPA was increased by 394% and DHA was decreased by 3%. Thus, it is plausible that there may be a threshold effect between endogenous ω-3 PUFAs levels and cardiovascular benefits. This mechanism may explain the lack of clinical benefit in the STRENGTH trial (67) since the level of EPA was not high enough to render significant outcomes. It is supposed that people who consume more fish on a regular basis and therefore have higher baseline ω-3 PUFAs level, such as those in Japan, may need lower doses to achieve the clinical benefit, whereas people who regularly consume western diet or less fish on average may need higher doses to reach the same therapeutic effect.

The imparity of placebos in the REDUCE-IT trial (mineral oil) (66) and the STRENGTH trial (corn oil) (67) may also play a role in explaining the controversial results. Unlike corn oil, mineral oil has major side effects such as reduction in statin absorption and proinflammatory effect. In addition, it is reported that different placebos may have different effects on some important biomarkers, e.g., triglycerides, LDL-C, and CRP (82, 84). Hence, many researchers attribute the inconsistent outcomes of ω-3 PUFAs partially to the influence of different placebos.

### Fish Oil and Arrhythmia

#### Fish Oil in Atrial Fibrillation

Earlier studies regarding the effect of ω-3 PUFAs on the prevention of post-operative atrial fibrillation (POAF) following cardiac surgery (CS) showed mixed results (85–91). In 2005, Leonardo calò et al. first reported results from an RCT including 160 patients with supplement ω-3 PUFAs 2 g/day (in the average ratio of EPA/DHA 1:2) for at least 5 days before CABG surgery until discharge (92). The results showed that POAF developed significantly less in the ω-3 PUFAs group (15.2%) than that in the control group (33.3%) (*p* = 0.013) (92). However, the OPERA trial obtained different conclusions. This large global multicenter trial enrolled 1,516 patients scheduled for CS (51.8% valvular surgery) (93). The experimental group received ω-3 PUFAs with pre-operative loading of 10 g over 3–5 days (or 8 g over 2 days) (each 1 g capsule contained EPA 465 mg and DHA 375 mg) followed post-operatively by 2 g/d until discharge or post-operative day 10 (93). The results of the OPERA trial showed that ω-3 PUFAs did not reduce the risk of POAF (30.7% in placebo group vs. 30.0% in ω-3 PUFAs group; OR: 0.96, 95% CI = 0.77–1.20; *P* = 0.74). Subsequent meta-analyses may provide a possible explanation for such controversial results. Wang et al. conducted a meta-analysis of 14 studies with 3,570 patients undergoing CS. It revealed that ω-3 PUFAs reduced the incidence of POAF (RR: 0.84; 95% CI = 0.73–0.98, *P* = 0.03) and this protective effect can be influenced by EPA/DHA ratio, i.e., an EPA/DHA ratio <1 indicated more favorable significance (RR: 0.51; 95% CI = 0.36–0.73, *P* = 0.0003). Moreover, ω-3 PUFAs seemed to only reduce the risk of POAF among patients undergoing CABG (RR: 0.68; 95 CI% = 0.47–0.97, *P* = 0.03) rather than other types of CS (94).

The ω-3 PUFAs supplement appears not to reduce AF recurrence in patients with known AF. Previous RCTs showed treatment with ω-3 PUFAs 4 g/day for 6 months did not reduce the recurrence of AF (95, 96). Other RCTs either raising the loading dose up to 8 g/d (97) or prolonging the follow-up up to 1 year (98), still found no significant results. Another study suggested that the time point to implement ω-3 PUFAs treatment before cardioversion may be important for the recurrence of AF (99). Nodari S conducted an RCT of 199 patients with persistent AF who were assigned to receive ω-3 PUFAs 2 g/d or placebo and then underwent cardioversion 4 weeks later after the treatment; at 1-year follow up, the rate of maintenance in sinus rhythm was significantly higher in the ω-3 PUFAs-treated patients compared with the placebo group (HR: 0.62; 95% CI = 0.52–0.72 and HR: 0.36; 95% CI = 0.26–0.46], respectively; *P* = 0.0001) (100, 101). A meta-analysis found similar results that by administering ω-3 PUFAs at least 4 weeks prior to cardioversion continuously, the recurrence rate of AF was significantly low (OR: 0.39; 95% CI = 0.25–0.61; *p* < 0.0001) in the ω-3 PUFAs group. Thus, current data suggested that, when considering ω-3 PUFAs, the treatment should be started at least 4 weeks before cardioversion, whereas initiating ω-3 PUFAs treatment <4 weeks before cardioversion (102) or even after cardioversion (103) were not found to reduce the risk of AF recurrence.

Intriguingly, several RCTs reported that high-dose ω-3 PUFAs supplementation even increased the risk of developing AF. Both the REDUCE-IT trial and the STRENGTH trial were conducted in patients with known CVDs who were treated with ω-3 PUFAs 4 g/d. The REDUCE-IT trial showed that patients randomized to ω-3 PUFAs group had more frequent AF events compared with placebo (3.1 vs. 2.1%, *P* = 0.004) (66). Similarly, in the STRENGTH study, patients treated with ω-3 PUFAs had an increased risk of new onset AF compared with those treated with corn oil (HR: 1.69; 95% CI = 1.29–2.21) (67). A recent meta-analysis of 38 RCTs including 1,49,051 patients showed that ω-3 PUFAs increased the risk of AF (RR: 1.26; 95% CI = 1.08–1.48) (104). It is also reported that EPA monotherapy was associated with a higher risk of AF (RR: 1.35; 95% CI = 1.10–1.66) (104). Most recently, Gencer et al. conducted a meta-analysis of 7 RCT including 81,210 patients, which demonstrated that a higher dose (>1 g/d) of ω-3 PUFAs was associated with an additionally increased risk of AF (HR 1.49; 95% CI = 1.04–2.15, *P* = 0.042) (105).

**Figure 5:**
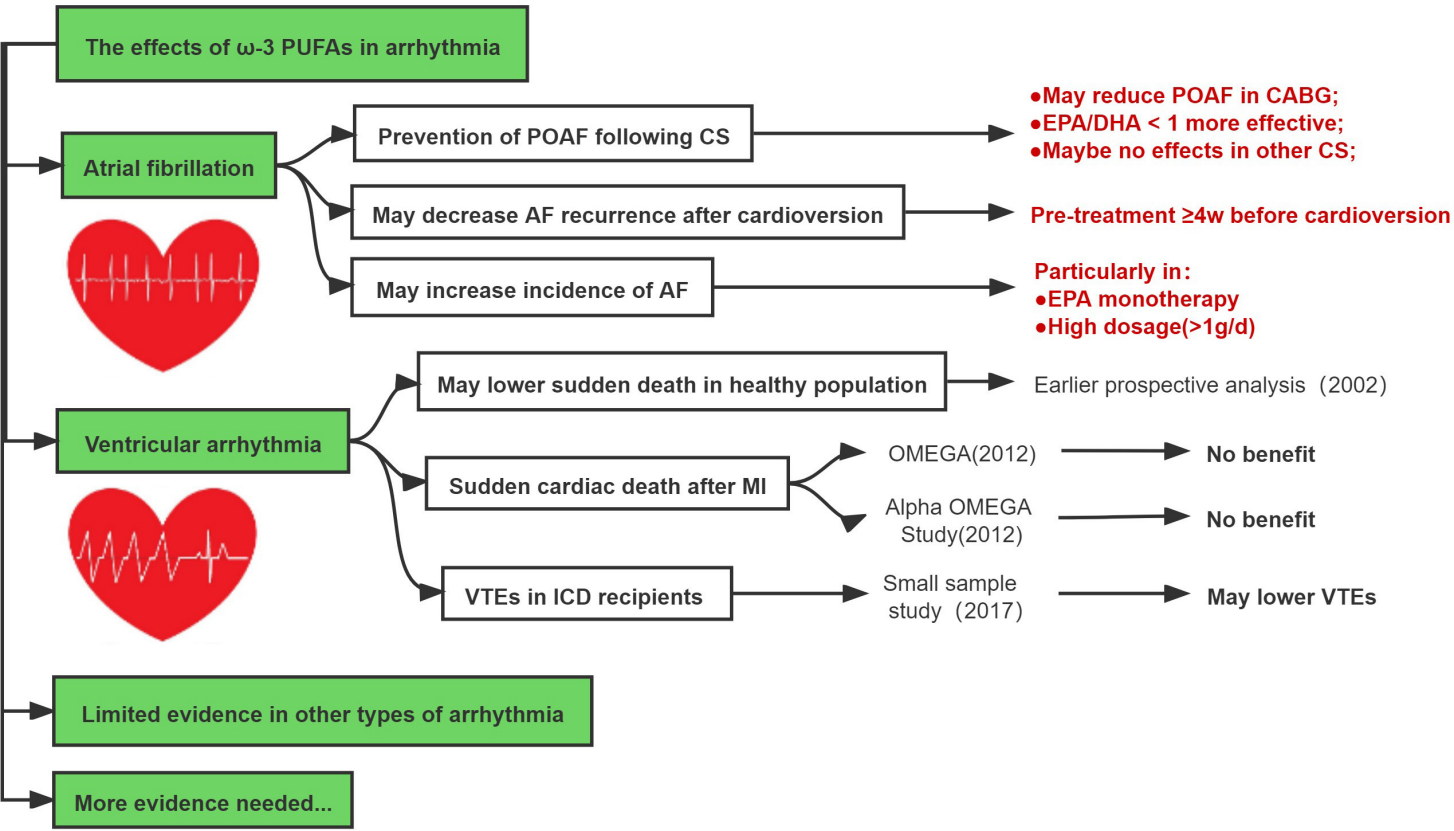
The effects of ω-3 PUFAs in atrial fibrillation and ventricular arrhythmia.

The current evidence suggests that ω-3 PUFAs seem effective in preventing POAF in patients undergoing CABG when EPA/DHA <1 during short-term follow-up and may reduce AF recurrence after cardioversion provided ω-3 PUFAs was continuously given at least 4 weeks before the cardioversion. However, high-dose ω-3 PUFAs may increase the risk of AF (Figure 5).

#### Fish Oil in Ventricular Arrhythmia

Several earlier retrospective and prospective studies have shown that ω-3 PUFAs supplementation may reduce the risk of ventricular tachycardia (VT) or ventricular fibrillation (VF) following MI, and reduce the risk of sudden death (76, 106–109). In 2002, Christine et al. conducted a prospective, nested case-control analysis among healthy men who were followed for up to 17 years (110). As compared with men whose blood level of ω-3 PUFAs were in the lowest quartile, the relative risk of sudden death was significantly lower among men with the level of ω-3 PUFAs in the third quartile (RR: 0.28; 95 CI% = 0.09–0.87) and the fourth quartile (RR: 0.19; 95 CI% = 0.05–0.71) (110).

On contrary, RCTs reported in 2010 (more contemporary era) including the OMEGA (111) and the Alpha Omega Study (78) failed to reveal the additional advantage of ω-3 PUFAs in reducing sudden cardiac death after MI. In 2017, a double-blind crossover placebo-controlled study evaluated the effects of ω-3 PUFAs on ventricular arrhythmias episodes (VTEs) in patients with implantable cardioverter defibrillator (ICD) due to ischemic cardiomyopathy (112). Among patients who received 3.6 g of EPA/DHA or placebo for 6 months, the mean number of VTEs was significantly lower in ω-3 PUFAs group vs. placebo (1.7 vs. 5.6; *p* = 0.035), despite the similar rate of ICD shocks between both groups (0.11 ± 0.6 vs. 0.10 ± 0.4, *p* = not significant, respectively) (112).

In summary, although several experimental studies and earlier clinical studies suggested that ω-3 PUFAs may be associated with reduced risk of ventricular arrhythmias, however, these favorable effects seemed not to be reproducible in recent prospective or retrospective studies, and the clinical value of ω-3 PUFAs in preventing ventricular arrhythmias in the contemporary era remains to be investigated (Figure 5).

### Fish Oil and HF

**Figure 6:**
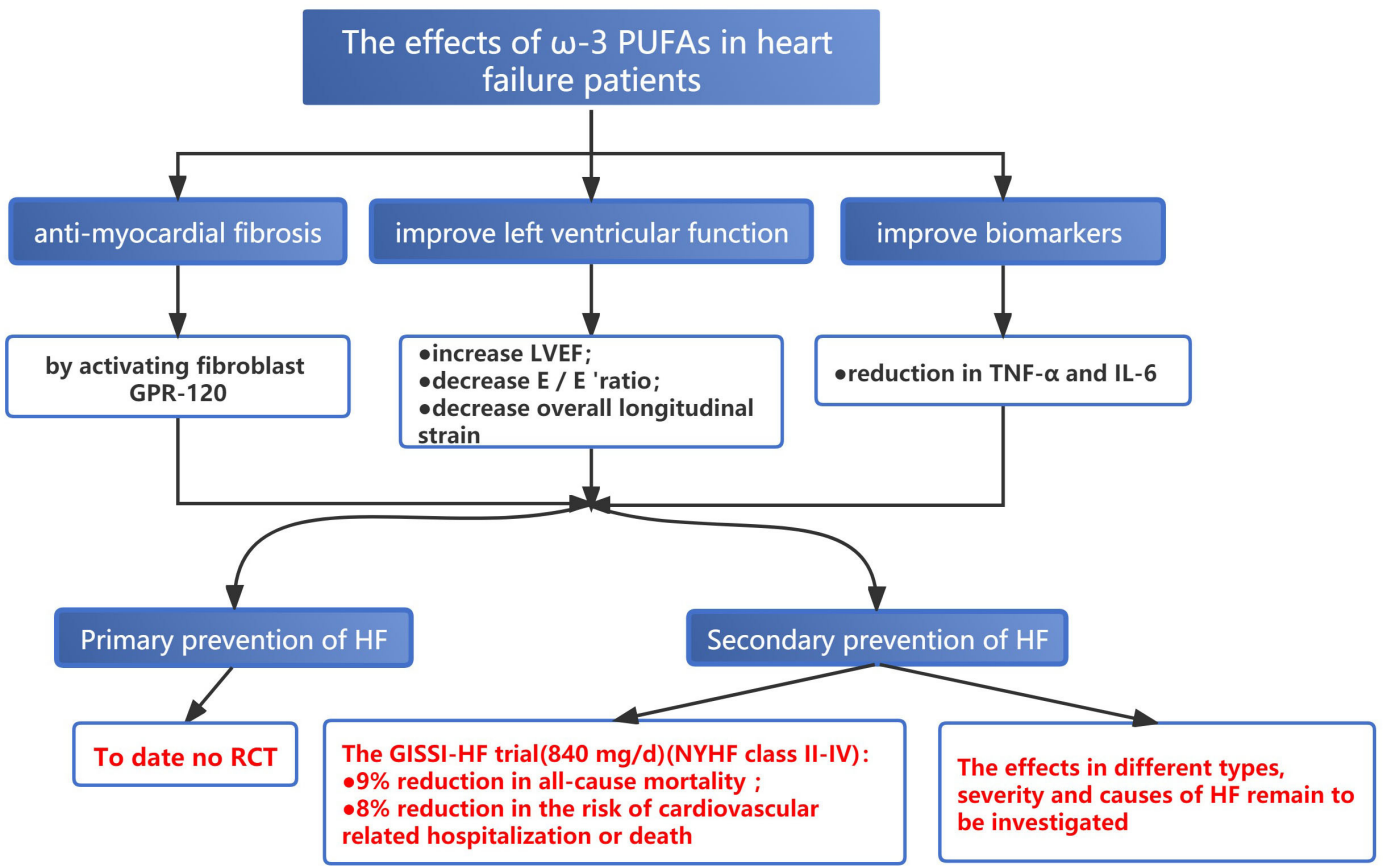
The effects of ω-3 PUFAs in heart failure.

Both basic and clinical studies indicated that adequate EPA intake, especially in the context of the low-fat diet, may antagonize cardiac fibrosis by activating fibroblast G-protein-coupled receptor 120 (GPR-120) (113, 114). To date, no published RCTs have assessed the effect of ω-3 PUFAs supplement on the primary prevention of HF. For the second prevention, despite inconsistent findings, most of the studies demonstrated the protective role of ω-3 PUFAs in the secondary prevention of HF (115–118). The GISSI-HF trial conducted in 2008 enrolled 6,875 patients with HF of different etiologies (New York Heart function class II-IV) to receive 840 mg/d EPA+DHA or placebo, respectively. After a mean follow-up of 3.9 years, ω-3 PUFAs supplementation reduced the risk of all-cause mortality by 9% (RR: 0.91; 95% CI = 0.833–0.998; *P* = 0.041) and the risk of cardiovascular-related hospitalization or death by 8% (RR: 0.92; 95% CI = 0.849–0.999; *P* = 0.009) (119). But the study did not further investigate whether the effect of ω-3 PUFAs varied among different specific types, severity, and causes of HF. A meta-analysis of 7 RCTs involving biomarkers for HF showed that TNF-α and IL-6 were significantly decreased after supplementing fish oil and greater reduction can be achieved in patients taking fish oil of a higher dose (over 1 g/d) or for a longer duration (over 4 months) (120). Evangelos et al. enrolled 31 patients with ischemic HF, and ω-3 PUFAs (2 g/d for a total of 8 weeks) were administered in the experimental group after a 6-week washout period (121). The results showed that short-term treatment with ω-3 PUFAs improved inflammatory, fibrotic status, and endothelial function, along with increased left ventricular ejection fraction (LVEF) (4.7 vs. 1.7%); decreased E/E 'ratio (early ventricular filling to early mitral annulus velocities) (9.47 vs. 2.1% reduction), and overall longitudinal strain (10.6 vs. 2.3% reduction) (Figure 6) (121).

## The Adverse Effects of Fish Oil

**Figure 7:**
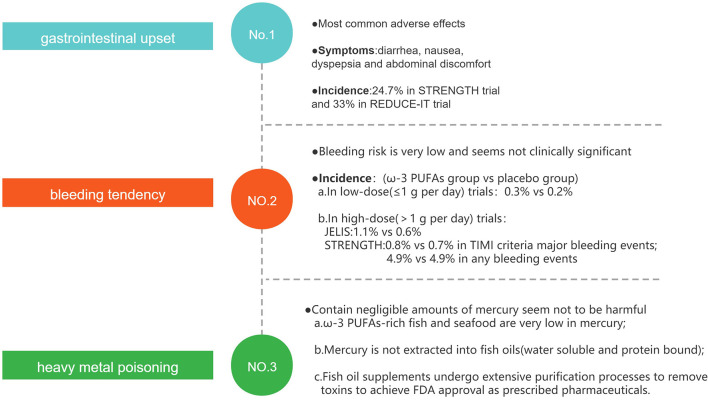
The potential adverse effects of fish oil.

The most commonly observed adverse effects of ω-3 PUFAs supplementation are gastrointestinal upset, bleeding tendency, and heavy metal poisoning (Figure 7), but those adverse effects tend to be mild or self-limited, and the supplements of fish oil are generally well-tolerated (122).

Gastrointestinal disorders such as diarrhea, nausea, dyspepsia, and abdominal discomfort may be the most common adverse effects. The rate of gastrointestinal adverse events reported in STRENGTH (67) and REDUCE-IT (66) trials was 24.7 and 33%, respectively. These results suggest that about a quarter of patients taking fish oil will experience gastrointestinal adverse events, which may limit the use of ω-3 PUFAs in patients with digestive disorders.

Due to the anti-thrombotic effect, another well-known adverse effect of fish oil is bleeding tendency. In low-dose ω-3 PUFAs (≤1 g per day) trials, the rate of bleeding events was similar in the ω-3 PUFAs group and the placebo group (0.3 vs. 0.2%) (59). In high-dose ω-3 PUFAs (>1 g per day) trials, overall hemorrhage was greater with EPA in the JELIS trial (1.1 vs. 0.6% no intervention) though patients with a history of hemorrhage were excluded (65). In the STRENGTH trial, both TIMI criteria major bleeding events (0.8 vs. 0.7%) and any bleeding events (4.9 vs. 4.9%) were similar, and 71% of participants were on one or more antiplatelet agents at baseline (67). This clinical evidence suggests that in general, the increased bleeding-risk of ω-3 PUFAs is very mild and not of clinical significance, even when antiplatelet therapy is concurrently administered (123).

Heavy metal poisoning, such as methyl mercury, is another concern when consuming fish oil. Three facts may suggest that fish oil supplements contain negligible amounts of mercury, and they may not be harmful to human health. First, commonly consumed ω-3 PUFAs–rich fish and seafood, such as salmon, shrimp, sardines, trout, herring, and oysters, are very low in mercury; and, mercury is water soluble and protein bound and is, therefore, not extracted into fish oils (124). Finally, selected fish oil supplements undergo extensive purification processes to remove environmental and other toxins, and prescribed fish oil preparations undergo even more rigorous regulatory processes to achieve the United States Food and Drug Administration (FDA) approval as prescribed pharmaceuticals (123, 125).

## Effect of ω-6 PUFAs/ω-3 PUFAs Ratio on CVDs

The ω-6 PUFAs and ω-3 PUFAs are generally considered to have beneficial health effects, but they have opposing effects on metabolic functions that might result in related pathological processes if the balance in the diet is altered (126). In general, metabolites of ω-6 PUFAs are proinflammatory, whereas metabolites of ω-3 PUFAs have anti-inflammatory, repairing, and protective effects. The ratio of the ω-6/ω-3PUFAs in the diet may determine the level of proinflammatory or anti-inflammatory proportion (127). There has been a growing interest in optimal ω-6/ω-3PUFAs ratio to improve the clinical outcomes, rather than in specific amounts of certain fatty acids. In the western diet, the estimated average ratio is approximately up to 20/1, with a ratio as high as 50/1 in South Asia (127). But what is the best proportion for human health? In most studies, ratios of 4–5/1 or less are recommended and are considered as appropriate dietary intake ratios (128, 129). Low ratios of ω-6/ω-3PUFAs (1.28–9.98) can downregulate the hepatic and aortic CRP expressions and reduce the aortic plaque lesions (130); it is also observed that infarct size after MI was significantly smaller in the 1/1 ratios group than that in the 5/1, or 20/1 ratios group (131); 1/1 ratio was associated with the lower atherosclerotic formation in mice, and the severity of atherosclerosis was increased as the ratio raised from 4/1 to 20/1 (132). These findings may shed new light on future studies.

## Conclusions

Previous basic researches have shown that ω-3 PUFAs may have favorable effects on anti-inflammation, anti-oxidation, improving endothelial function, antithrombosis, lowering blood lipids, improving left ventricular remodeling, stabilizing cellular electrical activity, and regulating autonomic nervous function.

In the clinical setting, ω-3 PUFAs may prevent atherosclerosis, stabilize, or reduce plaque. As secondary prevention of CHD, ω-3 PUFAs (particularly with high-dose and/or long-term treatment) may be associated with improved clinical outcomes among patients with suboptimal medical treatment, however, the additional merit from ω-3 PUFAs among patients already receiving optimal treatment remains to be determined. More randomized controlled studies are warranted to further evaluate the effects of EPA, DHA, or optimal combination of EPA+DHA on cardiovascular outcomes and the related mechanisms.

Consumption of ω-3 PUFAs seems to be associated with a reduction in BP among the different hypertensive populations and may lower the risk of developing hypertension in normotensive subjects; notably, the extension of BP lowering effect seems to be mild and appears to be dependent on the baseline BP.

ω-3 PUFAs supplement may prevent POAF in patients undergoing CABG or may reduce AF recurrence after cardioversion; however, high-dose ω-3 PUFAs may increase the risk of AF development. Although earlier clinical studies suggested that ω-3 PUFAs may be associated with reduced risk of ventricular arrhythmias, mortality, and hospitalization among known patients with HF; however, the evidence remains limited and seem not to be reproducible in large clinical studies in the contemporary medical setting.

## Author Contributions

SC, JL, and QX conceived the study and carried out the first draft of the manuscript and its major revisions. SC, ZL, and YY revised and edited the manuscript. SC and ZL are co-mentors for this dissertation. All authors contributed to the article and approved the submitted version.

## Funding

This work was partly funded by the fifth batch of Young and Middle-aged High-level Medical Talents Training Project of Chongqing Municipal Health Commission (Grant No: 2019-181, to ZL) and Kuanren Talents Program of the Second Affiliated Hospital of Chongqing Medical University, Chongqing, China [Grant No: (2020)7, to ZL].

## Conflict of Interest

The authors declare that the research was conducted in the absence of any commercial or financial relationships that could be construed as a potential conflict of interest.

## Publisher's Note

All claims expressed in this article are solely those of the authors and do not necessarily represent those of their affiliated organizations, or those of the publisher, the editors and the reviewers. Any product that may be evaluated in this article, or claim that may be made by its manufacturer, is not guaranteed or endorsed by the publisher.
